# Dr. Byron L. Craft

**Published:** 1916-12

**Authors:** 


**Dr. Byron L. Craft,** Buffalo 1893, of Newark, N. J., died in East Elmira, N. Y., Sept. 17, aged 48. Death occurred suddenly and was ascribed to heart disease. He formerly resided in Baltimore.

